# Nasogastric tube insertion length measurement and tip verification in adults: a narrative review

**DOI:** 10.1186/s13054-023-04611-6

**Published:** 2023-08-18

**Authors:** Kurt Boeykens, Tom Holvoet, Ivo Duysburgh

**Affiliations:** Nutrition Support Team, VITAZ Hospital, Moerlandstraat 1, 9100 Sint-Niklaas, Belgium

**Keywords:** Nasogastric tube, Tube feeding, Enteral nutrition, Gastrointestinal intubation, Patient safety

## Abstract

Nasogastric feeding tube insertion is a common but invasive procedure most often blindly placed by nurses in acute and chronic care settings. Although usually not harmful, serious and fatal complications with misplacement still occur and variation in practice still exists. These tubes can be used for drainage or administration of fluids, drugs and/or enteral feeding. During blind insertion, it is important to achieve correct tip position of the tube ideally reaching the body of the stomach. If the insertion length is too short, the tip and/or distal side-openings at the end of the tube can be located in the esophagus increasing the risk of aspiration (pneumonia). Conversely, when the insertion length is too long, the tube might kink in the stomach, curl upwards into the esophagus or enter the duodenum. Studies have demonstrated that the most frequently used technique to determine insertion length (the nose–earlobe–xiphoid method) is too short a distance; new safer methods should be used and further more robust evidence is needed. After blind placement, verifying correct gastric tip positioning is of major importance to avoid serious and sometimes lethal complications.

## Background

Placement of a nasogastric tube (NGT) is a blind technique where the tube is inserted through the nostril, along the nasopharynx, through the esophagus and into the stomach. In the intensive care unit (ICU), NGT should be used as the standard approach for enteral feeding [1]. The tube can also be used to drain contents of the stomach or to administrate drugs or fluids. Approximately 10 million nasogastric tubes in Europe and 1.2 million in the United States are placed annually [2].

It has been stated that a NGT is correctly positioned when the tip is located between 3 and 10 cm under the lower esophageal sphincter although this is subjective since tilting the head forward can add 5 cm to the length, so 10 cm below the left hemi-diaphragm would be a safer margin [3–5]. Inaccurate placement of the tube can lead to complications. Overestimation of the insertion length can cause coiling of the tube inside the stomach, upward migration back into the esophagus or downwards into the duodenum. The latter can lead to dumping syndrome when using bolus feeding. Underestimation of the insertion length can lead to tube feeding remaining in the esophagus, increasing risk of tube feeding formula aspiration. Additionally, NGT placement can also provide symptomatic relief and decompression in case of small bowel obstruction, gastric outlet obstruction (e.g., in case of severe pancreatitis) or ileus. Adequate drainage, which will minimize the risk of vomiting and/or aspiration, also depends on the proper depth of the inserted tube and correct location of the tip and distal side-openings. Measuring gastric residual volumes as surrogate parameter to define gastric emptying and to increase enteral nutrition feeding rate is frequently applied on the ICU. ‘(In)correct position and lateral side openings of the NGT can influence this measurement [6].

Correct placement of a NGT is thus important but correct assessment of the gastric tip positioning remains challenging [7, 8]. In an ICU study (740 feeding tube placements), 14 NGT’s were inserted into the tracheopulmonary system (13 patients were intubated) and two patients died because of complications directly related to the feeding tube placement. Malposition of the feeding tube was not predictable from clinical signs and auscultation [9]. Several techniques are currently available in the literature. This narrative review aims to summarize and discuss the existing evidence (methods or techniques) for nasogastric tube insertion length measurement and tube tip verification.

## Methods of tube insertion length measurement

### The nose–earlobe–xiphoid method (NEX)

**Fig. 1:**
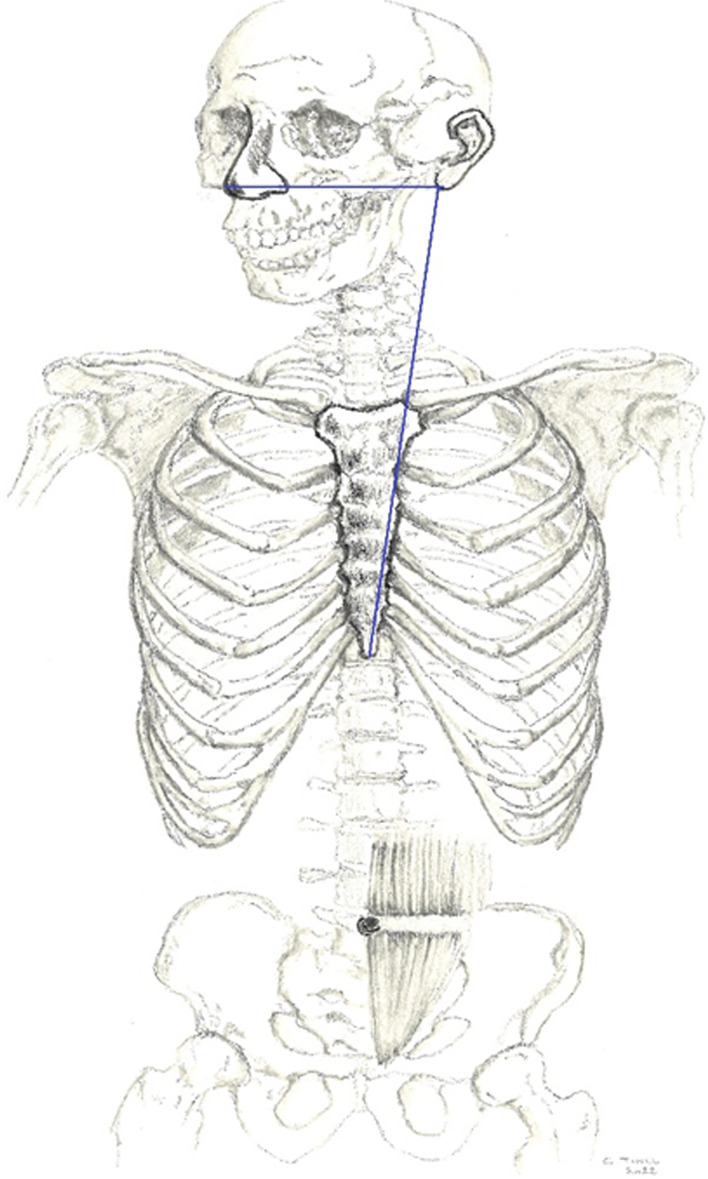
The NEX is the distance from nose to earlobe to xiphisternum (skeleton drawing: Chis Tinel)

The nose–earlobe–xiphoid method (NEX) was proposed in 1951 [10]. The NEX is the distance from nose to earlobe to xiphisternum (see Fig. 1). Because the xiphisternum is more difficult to locate, it is better to measure it in the opposite direction: xiphisternum to ear to nose (XEN) [4]. A recent meta-analysis, summarizing all available evidence from 1951 until 2022, showed accuracy of the NEX method at only ≤ 72.4% [11]. Despite the low accuracy rate, the NEX method still remains the most widely used method for tube insertion in adults [12, 13].

### The XEN + 10 cm

**Fig. 2:**
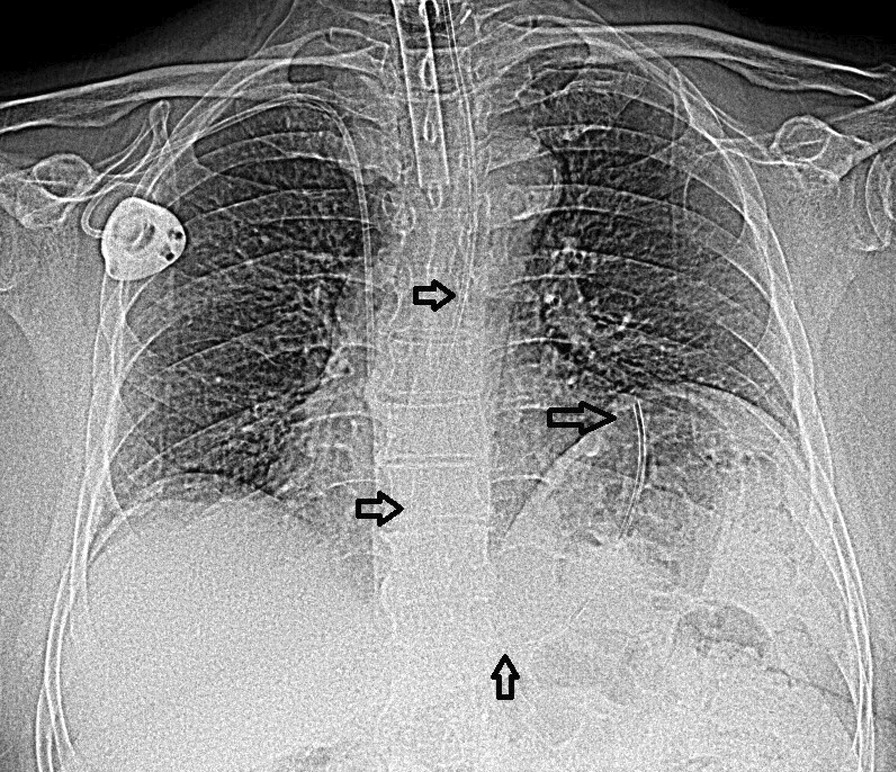
This small-bore NGT (French 10) is placed on NEX distance (57 cm) and located with the tip in the fundus. XEN + 10 cm (67 cm) could potentially result in further coiling or kinking of the NGT

**Fig. 3:**
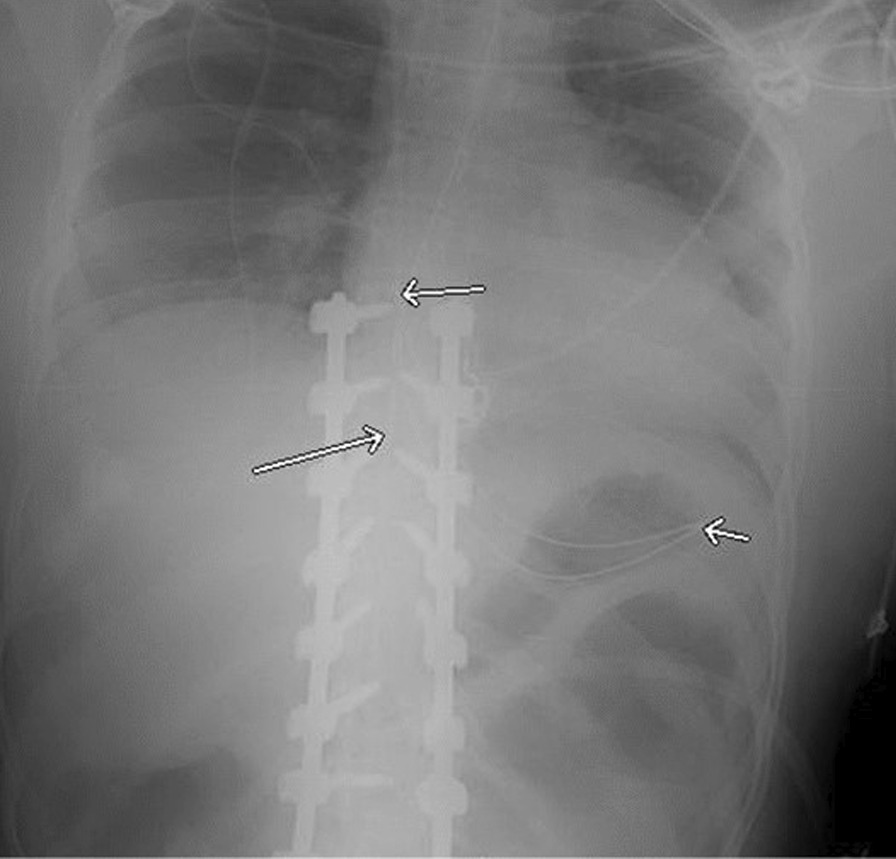
This tube for drainage is placed on XEN + 10 distance (69 cm) but curls in the stomach and migrates upwards ending with the tip in the distal esophagus

In a prospective single-center observational study, Taylor et al. used electromagnet-guided tube placement in 200 intensive care patients to correctly estimate the tube length from the nose to its optimal position in the gastric body. In this study, ideal nasogastric tube positioning was suggested at XEN + 10 cm. Although guided tube placement can eliminate risk of transpyloric or esophageal placement, blind placement of XEN + 10 cm might on the one hand increase the risk of transpyloric positioning (and dumping syndrome if bolus feeding is used) and on the other hand, tube recoiling into the esophagus. Indeed, if the NG tube tip hits the greater curvature of the stomach, the inserted tube length will determine how far it coils back (see Figs. 2 and 3). The longer the inserted length, the greater the risk that the tip will end up in the (distal) esophagus (see Fig. 3) but this possible association has not been studied yet [5, 11, 14].

### The corrected NEX (CoNEX) method

A recent randomized controlled study (RCT) in 183 ICU patients showed that in > 20% of all patients insertion length underestimated the required depth for correct positioning when using both the NEX and Hanson method ((NEX × 0.38696) + 30.37 cm) [15, 16]. This is in line with 19–23% of esophageal placement found on X-ray in another study after end of procedure [17]. Based on these findings, a ‘corrected nose–earlobe–xiphoid distance’ (CoNEX) method was developed using next corrected Hanson formula: ((NEX × 0.38696) + 30.37 + 6 cm). In a prospective study of 218 ICU patients, the CoNEX showed a correct tip position (defined as > 3 cm in the stomach) in all patients while no tubes were seen migrating back into the esophagus with 94% ≥ 6 cm under the left hemi-diaphragm. Transpyloric tip position was only seen in one patient (0.5%). The overall chance of successfully obtaining gastric aspirate within 2 h after NGT placement was 77.9% compared to ± 55% in both groups in the above-mentioned RCT using NEX or Hanson method [18]. For easy application of the CoNEX method in clinical practice a conversion table can be used (see Table 1).

**Table 1:** NEX distances (to be measured) and corresponding CoNEX insertion lengths

NEX	CoNEX	NEX	CoNEX	NEX	CoNEX
40	52	50	56	60	60
41	52	51	56	61	60
42	53	52	56	62	60
43	53	53	57	63	61
44	53	54	57	64	61
45	54	55	58	65	62
46	54	56	58	66	62
47	55	57	58	67	62
48	55	58	59	68	63
49	55	59	59	69	63

## Methods of tube placement verification

### Radiographic confirmation

In guidelines and ICU alerts there is general consensus that a properly obtained and interpreted X-ray is the gold standard to distinguish between gastric and pulmonary placement [19–21]. However, in 2011 in the United Kingdom, a safety alert/report by the former National Patient Safety Agency showed that misread X-ray position checks caused 12/21(57%) of gastric tube-related deaths and 45/76 (59%) of tube-related complications [22]. Possible explanations are a low degree of expertise of the interpreter, poor radiopacity of the tube and low X-ray quality (e.g., inability to visualize key anatomy) [23, 24]. Radiology reports often do not include information regarding correct tip position, but on the other hand, the request form should be standardized and clearly state that the purpose of the X-ray is to establish the position of the NGT for feeding, fluid or medications [25, 26]. Other disadvantages of X-ray confirmation include waiting times, excess X-ray usage and availability (e.g., in care homes, rehabilitation centers and in the home care setting). It also can further lead to a delay in fluid, feed and drugs up to 8–9 h per tube placement and loss of 18.8% of feeding time per EN episode [27].

### The ‘whoosh test’ or air insufflation method

The ‘whoosh test’ consists of rapidly injecting air down a NGT while auscultating (listening for a ‘whooshing sound’) over the epigastrium. Although research has established the inadequacy of auscultation to determine proper tube placement, this method is still commonly practiced in ICU’s [28]. In 2014, 15 published case reports in children were reviewed; four children died because of mispositioned tubes using this method. The auscultatory bedside method failed to detect the mispositioned tubes in all seven cases in which it was used [29]. The auscultatory method lacks specificity (the probability that the test correctly indicates when the NG tube is placed outside the stomach), making it an unreliable method to assess correct tube placement [30]. However, injecting air without auscultating may identify a kinked tube and esophageal placement may be suspected if the air is “burped” back by the patient [31].

### pH testing

Testing the acidity of fluid aspirated from the stomach to verify NGT placement has been advocated for decennia. In differentiating between gastric and respiratory placement different pH cut-off points have been used: ≤ 4, ≤ 5.5, ≤ 5.9, ≤ 6.5, ≤ 7 and ≤ 7.9 [32]. The problem with a low cut-off point (e.g.,  ≤ 4) is a low sensitivity (more false negative results) but a high specificity and with a high cut-off point this is the opposite (e.g.,  ≤ 7.9). Two studies proposed a pH of ≤ 5 as a safe, reliable and practical cutoff in adults and children to avoid respiratory feeding and to keep feeding in the esophageus to a minimum [33, 34]. In situations when the reading is unclear or when gastric juices could have been aspirated into the lungs (e.g., during intubation), the pH could be misleading and re-testing or a secondary confirmation by X-ray is recommended. Colorimetric test strips require subjective interpretation, so it may be difficult to make accurate readings, especially between pH values of 5 and 6 [35, 36]. Technologies for determining feeding tube tip location based on pH sensors (a disposable tube-specific guidewire with a pH sensor at the tip connected to a handheld/portable system) or pH test strip readers can overcome that problem. However, overall 50% of end-of-procedure pH checks failed because the pH was above the accepted threshold [37, 38].

### Capnography and colorimetric capnometry

To prevent pneumothorax at 30 cm or 40 cm tube depth, high CO_2_ levels can be detected by colorimetric capnometry or capnography [38]. A recent systematic review revealed a low to very low certainty of evidence that both methods are potentially effective in differentiating between respiratory and nasogastric tube placement for critically ill adult patients [39]. Therefore, if using these devices this should likely not be the sole source of tube verification.

### Ultrasound or ultrasonography

Ultrasound (US) can be used to visualize the tube via both the neck and abdomen and can be performed at the bedside. Visualization of the tube in the stomach is interpreted as correct positioning. Injecting air or saline during visualization can help to detect the tip position in the stomach. A large systematic review and meta-analysis found 14 studies where in total 1812 patients were included. The results showed a pooled sensitivity of 0.96 (95% confidence interval [CI] 0.94–0.97) and a specificity of 0.91 (95% CI 0.85–0.96). The authors concluded that ultrasound is an efficient method for verifying nasogastric tube placement, although there is insufficient evidence to suggest that it can be used as a diagnostic tool for incorrect gastric tube placement [40]. US cannot identify the full tube path from nose to intestine (only visibility within the esophageus, pyloric area and early duodenum part-1) and also requires two operators [41]. Technical difficulties may also exist in obese, patients with, e.g., gas in a bowel loop in some patients with a laparotomy, open abdomen, abdominal wall defect or drainage [42].

### Camera technology

Another new promising technological innovation is a single-use, small-bore NG feeding tube with a miniature camera embedded in the distal end to aid in tube placement. This system allows trained clinicians in all cases to correctly identify anatomical markers (esophageus, trachea or stomach) during placement. Disadvantages could be intolerance of the camera tip during nasal passage in conscious patients and costs of the equipment and supplies [38, 42–44].

### Electromagnetic guidance

This real-time indirect visualization technique refers to a guided feeding tube placement system that uses an electromagnetic sensing device to show the relative path of the feeding tube during a placement procedure (Cortrak™). It allows the user to recognize inadvertent lung malposition as it occurs and it assists the user to correct the placement immediately, rather than waiting for radiograph confirmation. In a large study with a total of 6290 feeding tube placements in 4239 patients, 68 lung placements were avoided in 2015 by recognizing proximal pulmonary deviation [45]. However, team experience seems a crucial prerequisite to safely use the technology. Looking at a database with adverse events, fifty-four adverse events occurred during a period of ten years’ time (2006–2016). Almost all events (98%) involved feeding tube placement in the lungs. Moreover, lung complications included pneumothorax (77%) and pneumonitis (21%) and death occurred in 17% of lung placements. Clinicians failed to recognize adverse events in 89% of insertion tracings reviewed [46]. So clinicians require specialized training and experience to develop competency in using this device. In centers with lowest complication rates, new operators need 50–75 supervisions [38].

Advantages and disadvantages of nasogastric tube insertion length measurement methods and (in)direct tube tip visualization techniques are summarized in Table 2.

**Table 2:** Nasogastric insertion length measurement methods and tube tip visualization techniques

	Advantages	Disadvantages
**Insertion length measurement**		
NEX		Not reliable Risk of over- and underestimation
XEN + 10 cm	Reduces risk of esophageal placement	Risk of overestimation Risk of recoiling
CoNEX	Reduces risk of esophageal placement Very low risk of overestimation Higher chance for gastric aspirate	No multicenter study No clinical efficacy study Based on limited data
**Tip position verification**		
Radiographic confirmation	Gold standard if well interpreted and properly obtained	Misinterpretation possible Reporting of tube location can be lacking Repeated and prolonged delays to feed, drugs and fluid possible
Air insufflation method or ‘whoosh test’		Not reliable Lacks specificity
pH testing	Safe and feasible bedside method with pH cut-off ≤ 5 Can also be used outside the hospital	Inter-observer variability in reading pH test result With pH cut-off ≤ 5 there is still a very small risk of feeding in the esophageus Ability to obtain an aspirate with fine-bore tubes
Capnography and Colorimetric Capnometry	Useful to detect trachea placement during insertion process (tube depth 30 cm) and avoid deep lung penetration	False positive observations were reported Need for an end-of-procedure tube position check
Ultrasound	High success rate without any complication	Requires 2 operators Not applicable or difficult in some patients (e.g., abdominal wall defects, morbid obese, open abdomen)
Camera technology	Correctly identifies internal anatomy (gastric/pulmonary) and end-tube position Pre-empts lung trauma	Need for trained clinicians Cost of the equipment
Electromagnetic guidance	In expertized centers high agreement with X-ray Early detection of respiratory placement	Need for long training period Cost of the equipment and supplies

## Conclusions

Two safety issues are important in the placement of NGT’s: correct insertion length and tip position. The NEX method should no longer be used mainly for the risk of underestimation. XEN + 10 cm and CoNEX can overcome this shortcoming and should currently be used in clinical practice. But further studies with blind tube placement need to demonstrate maximal safety before incorporation into guidelines. Radiologic confirmation of blindly inserted nasogastric feeding tubes remains the golden standard although it has some drawbacks such as possible misinterpretation, feeding delay, X-ray exposure and costs. The air insufflation method is unsafe and should never be used. pH-measurement with a pH cut-off point of 5 seems currently the most practical and feasible bedside method but correct use, training and interpretation are required. New innovative methods such as pH sensors can overcome inter-observer variability with pH strips, but their extra value should be tested in randomized controlled trials. Capnography and colorimetric capnometry are alternatives to detect pulmonary placement and could reduce the risk of pneumothorax. Finally, ultrasound, electromagnetic guidance and camera technology could decrease the number of X-rays, earlier detect respiratory placement and reduce the time to (re)start feeding. But for safe use you need conclusive results and well-trained clinicians. Overall, cost-benefits of each method should be balanced taking into account additional healthcare costs due to complications, repeated investigations and misplacements. In conclusion, no method for determing tube insertion length or tip position verification are completely safe and warrant further investigation.

## Data Availability

Not applicable.

## Abbreviations

UICU: Intensive care unit
NEX: Nose–earlobe–xiphoid
XEN: Xiphoid–nose–earlobe
LES: Lower esophageal sphincter
CoNEX: Corrected NEX
RCT: Randomized controlled trial
UK: United Kingdom
NGT(’s): Nasogastric tube(s)
US: Ultrasound
CI: Confidence interval
CO_2_: Carbon dioxide
